# Inhibition of the CXCR4/PLC Signaling Increases Dexamethasone-Induced Sensitivity by Activating the Mitochondrial Apoptotic Pathway in B-Cell Acute Lymphoblastic Leukemia

**DOI:** 10.3390/ijms26083489

**Published:** 2025-04-08

**Authors:** Souleymane Abdoul-Azize, Jean-Pierre Vannier, Pascale Schneider

**Affiliations:** 1 University Rouen Normandie, Inserm, UMR 1234, 76000 Rouen, France; 2 Department of Pediatric Immuno-Hemato-Oncology, University Hospital, 76000 Rouen, France

**Keywords:** acute lymphoblastic leukemia, dexamethasone, PLC inhibitor, CXCR4 inhibitor, mitochondrial pathway

## Abstract

Understanding the mechanisms underlying glucocorticoid (GC) resistance in B-cell acute lymphoblastic leukemia (B-ALL) is essential to improve survival rates in relapsed children. We previously showed that GCs paradoxically induced their own resistance in B-ALL through CXCR4/PLC signaling, and that the inhibition of this pathway significantly reverses GC resistance in B-ALL cells and improves survival of GC-treated NSG mice in vivo. Here, we sought to determine whether the enhancement of GC sensitivity via inhibition of the CXCR4/PLC axis is associated with disruption of the mitochondrial pathway. Analysis of our previous transcriptomic data revealed that in B-ALL, the PLC inhibitor U73122 compromised multiple metabolic pathways related to metabolic reprogramming, mitochondrial function, and oxidative stress. Inhibition of PLC with U73122, protein kinase C with GF109203X, or CXCR4 with AMD3100 significantly potentiated dexamethasone (Dex)-induced mitochondrial membrane potential depolarization, reactive oxygen species production, cytochrome c release, caspase-3 activation, and decreased O_2_ consumption in B-ALL cells. These observations were also confirmed after Dex treatment in a B-ALL Nalm-6 cell line transfected with CXCR4 small interfering RNA. Moreover, co-treatment with Dex and CXCR4, PKC, or PLC inhibitors increased the levels of the pro-apoptotic protein BIM (BCL-2 interacting mediator of cell death) and, consequently, promoted the cell death process. Together, these findings suggest that the CXCR4/PLC axis reduces Dex efficacy by limiting mitochondrial apoptotic activity.

## 1. Introduction

Glucocorticoids (GCs) are the most commonly used drugs in the treatment of acute lymphoblastic leukemia (ALL), the most common childhood cancer [1,2,3]. Despite significant improvements in treatment outcomes, 15–20% of children relapse and become resistant to chemotherapy, starting from the induction phase that includes GC monotherapy [4,5,6,7]. Thus, a lack of initial response to GCs is predictive of treatment failure and, consequently, of poor overall disease prognosis [8,9,10]. This underscores the need to understand the mechanisms behind primary resistance to GCs. GCs exert their effects through cytoplasmic glucocorticoid receptors (GRs), triggering their translocation to the nucleus and initiating a transcriptional program that induces apoptosis in lymphoid cells. However, in ALL, GCs may paradoxically promote resistance [1,11,12]. In T-ALL, for instance, GCs can increase IL-7 receptor (IL-7R) expression, leading to the upregulation of the pro-survival protein BCL-2 [13]. While IL-7R is essential for normal T- and B-cell development [13,14,15], this mechanism is specific to T-ALL, as in B-ALL, GCs repress IL-7R expression [16]. In B-ALL, intracellular Ca^2+^ signaling plays a crucial role in the resistance to GC treatment, and Ca^2+^ chelation enhances GC sensitivity [17,18]. Investigating this pathway activated by GCs in B-ALL therapy is therefore crucial.

In a previous study, we demonstrated that GCs paradoxically induced their own resistance in B-ALL through CXCR4/PLC signaling [19]. This study suggested that during treatment, the GC dexamethasone (Dex) activated CXCR4 signaling, leading to the activation of PLC-dependent pathways, which increased intracellular Ca^2+^ and activated protein kinase C (PKC). These pathways reduced the anticancer efficacy of Dex and contributed to resistance in B-ALL. Inhibition of the CXCR4 receptor or the PLC pathway improved survival in Dex-treated NSG mice (NOD SCID gamma mice) and restored sensitivity in Dex-resistant B-ALL cell lines and patient-derived cells [19]. However, whether this improvement in GC sensitivity by inhibiting the CXCR4/PLC axis interferes with the mitochondrial pathway remains unclear.

In this work, we aimed to determine whether inhibition of the CXCR4/PLC axis, which improves sensitivity to GCs, is associated with disruption of the mitochondrial pathway. Our findings revealed that inhibitors of CXCR4/PLC signaling in combination with Dex produced an additive/synergistic effect on mitochondrial apoptotic activity in B-ALL. This effect is achieved by activating apoptosis mediators such as the BH3-only pro-apoptotic protein BIM, modulating the mitochondrial membrane potential, producing reactive oxygen species, releasing cytochrome c, activating caspase-3 activity and decreasing oxygen (O_2_) consumption. In conclusion, this study enhanced the understanding of the mechanisms by which inhibition of the CXCR4/PLC pathway improves the efficacy of Dex in the treatment of B-ALL and overcomes resistance. This approach could offer a new strategy for improving cancer therapy and patient survival.

## 2. Results

### 2.1. PLC Inhibition Is Associated with Dysregulated Metabolic and Mitochondrial Pathways in B-ALL Cells

**Figure 1:**
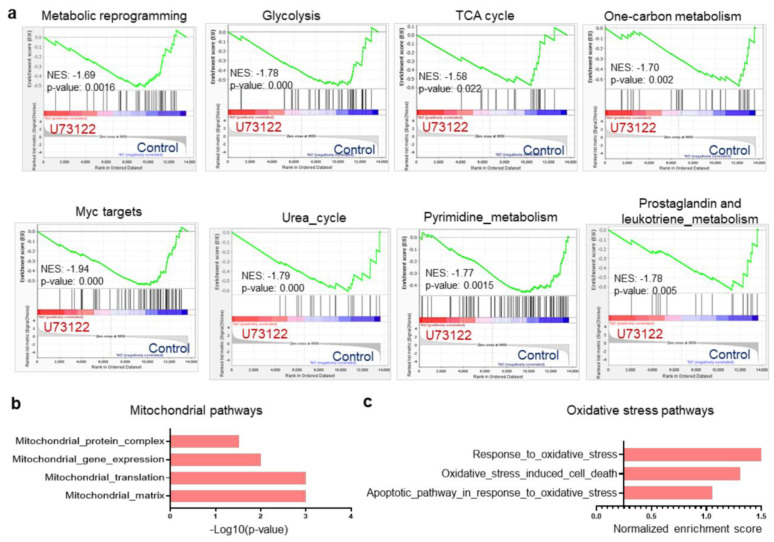
Inhibition of the PLC pathway dysregulates several metabolic pathways in Dex-resistant Nalm-6 B-ALL cells. (**a**) Gene set enrichment analysis (GSEA) plots of RNA-Seq showing multiple metabolic pathway signatures comparing Ctr versus 1 µM U73122 in Dex-resistant Nalm-6 cells after 16 h of treatment. (**b**) Functional clustering of downregulated genes in 1 µM U73122-exposed- Dex-resistant Nalm-6 cells showing impaired mitochondrial gene expression and function. (**c**) Normalized enrichment scores of Ctr versus 1 µM U73122-treated Dex-resistant Nalm-6 cells showing upregulated response to oxidative stress-mediated cell death as determined by GSEA. False discovery rate (FDR)-adjusted *p*-value < 0.05, *n*  =  2.

In light of our recent finding that inhibition of CXCR4/PLC-mediated signaling enhances Dex sensitivity in B-ALL cells [19], we hypothesized that defective metabolism and mitochondrial pathways may be associated with this mechanism. Since metabolic reprogramming in cancer cells is linked to the development of drug resistance [20], we first analyzed our previous RNA-sequencing data (GSE214990) to investigate the metabolic processes-regulated PLC pathway inhibition with U73122 in a Dex-resistant B-ALL cell line. We performed gene set enrichment analyses (GSEA) of differentially expressed gene (DEG) sets, which revealed the downregulation of multiple metabolic pathways in U73122-treated cells compared to Ctr cells, including pathways related to metabolic reprogramming, glycolysis, the tricarboxylic acid (TCA) cycle, one-carbon metabolism, Myc targets, the urea cycle, pyrimidine metabolism, and prostaglandin and leukotriene metabolism (Figure 1a). U73122-dependent alterations in metabolic reprogramming also significantly downregulated mitochondrial function, as supported by GSEA using gene ontology analysis, with evidence such as mitochondrial protein complex, mitochondrial gene expression, mitochondrial translation, and mitochondrial matrix (Figure 1b). To maintain a selective advantage for chemoresistance and growth, chemoresistant cells can switch between glycolysis and mitochondrial oxidative phosphorylation (OXPHOS) [21]. Additionally, we found an enrichment of oxidative stress pathways in U73122-treated cells, such as cell response to oxidative stress, oxidative stress-induced cell death, and the apoptotic pathway in response to oxidative stress (Figure 1c). These results suggest that PLC inhibition facilitated cell apoptosis mediated by reactive oxygen species (ROS). Taken together, these findings indicate that PLC controls several metabolic pathways in B-ALL cells, including oxidative stress and mitochondrial function.

### 2.2. Inhibiting PLC Improves Dex Sensitivity in B-ALL Cells by Altering the Mitochondrial Pathway

**Figure 2:**
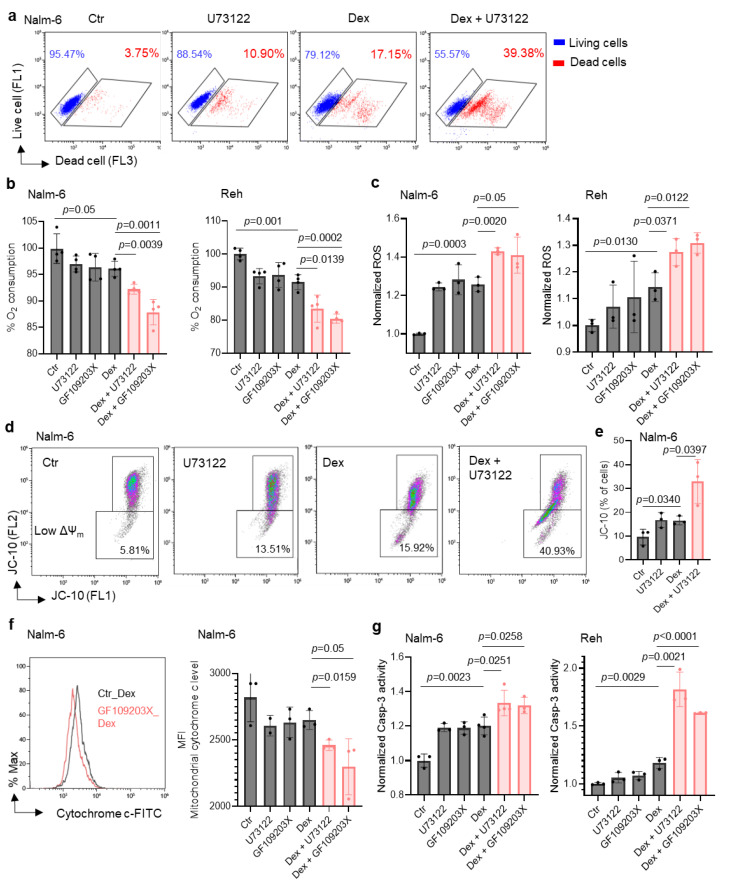
Co-treatment with Dex and U73122 or GF109203X markedly increases mitochondrial dysfunction in ALL cells. (**a**) Nalm-6 cells treated with 100 nM Dex or 1 μM U73122, alone or in combination, for 48 h. Alive and dead cells were determined using LIVE/DEAD^®^ staining kits and analyzed by flow cytometry. (**b**) Oxygen consumption rates in cells stimulated with 100 nM Dex and/or 1 μM U73122 or GF109203X for 24 h. (**c**) Intracellular reactive oxygen species (ROS) in cells stimulated with 100 nM Dex and/or 1 μM U73122 or GF109203X for 24 h. (**d**) Representative flow cytometry plots of Nalm-6 cells treated with 100 nM Dex and/or 1 μM U73122 for 24 h showing cells with mitochondria membrane potential change (ΔΨ_m_). (**e**) Quantification of cells with depolarized mitochondria from panel (**d**). (**f**) Representative flow cytometry plots and MFI quantification of mitochondrial cytochrome c level of cells treated with 100 nM Dex and 1 μM U73122 or GF109203X for 24 h. (**g**) Caspase-3 activity determined using Ac-DEVD-AFC as substrate after 24 h with 100 nM Dex and/or 1μM U73122 or 1μM GF109203X in Nalm-6 and Reh cells. Unpaired two-tailed Student’s *t* test, *n* = 3–4. Data are represented as mean ± SEM.

**Figure 3:**
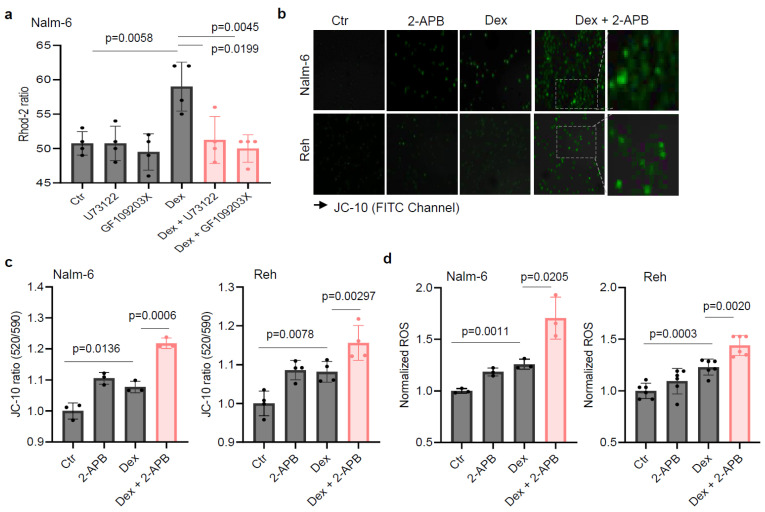
IP_3_-induced Ca^2+^ release inhibitor 2-APB increases Dex-induced mitochondrial membrane potential depolarization and reactive oxygen species production in ALL cells. (**a**) Mitochondrial Ca^2+^ in cells preincubated with either 10 µM U73122 or GF109203X during Rhod-2 loading before stimulation with 125 nM Dex in Ca^2+^-containing buffer (4 mM Ca^2+^). (**b**,**c**) Cells were exposed to 100 nM Dex in the absence or presence of 5 µM 2-APB for 24 h. (**b**) The images acquired with a Zeiss Axiovert 200 M fluorescence microscope after JC-10 staining using FITC channel. Scale bar: 10 µm. The fluorescence intensity for the mitochondrial membrane potential change was normalized to the Ctr in (**c**). (**d**) The cells were exposed to 100 nM Dex in the absence or presence of 5 µM 2-APB for 24 h. DHR 123 fluorescence intensity for ROS production was normalized to the Ctr. Unpaired two-tailed Student’s *t*-test, *n* = 3–6. Data are represented as mean ± SEM.

Based on the connection between the PLC pathway and metabolism, we hypothesized that PLC-mediated metabolic reprogramming regulation could contribute to B-ALL cell sensitivity to Dex. Using flow cytometry, we validated our previous finding that inhibiting PLC in vitro improves Dex sensitivity in B-ALL (Figure 2a) [19]. We then evaluated mitochondrial respiration by measuring the oxygen consumption rate (OCR) in B-ALL cells. As shown in Figure 2b, co-treatment with Dex and the PLC inhibitor (U73122) or PKC inhibitor (GF109203X) markedly decreased the OCR in ALL cell lines. We previously reported that combined treatment with the Ca^2+^ chelator (Bapta-AM) and Dex in B-ALL cells resulted in an increase in ROS production and mitochondrial membrane potential (Δψ_m_) dissipation [17]. Consistently, we observed a significant increase in reactive oxygen species (ROS) (Figure 2c) as well as Δψ_m_ depolarization (Figure 2d,e) in B-ALL cells co-treated with Dex and PLC and PKC inhibitors. ROS production and Δψ_m_ depolarization lead to the initiation of the mitochondria-mediated cell apoptosis cascade, involving cytochrome c release and caspase-3 activation. We next determined whether the effect of PLC pathway inhibition on Dex-induced cell death was associated with mitochondrial release of cytochrome c and increased caspase-3 activity. For this, we first analyzed mitochondrial cytochrome c levels in B-ALL cells stimulated with Dex in the presence or absence of the PLC and PKC inhibitors for 24 h using flow cytometry. Cells that release cytochrome c from the mitochondria to the cytoplasm show reduced staining mean fluorescence intensity (MFI). Dex-induced mitochondrial cytochrome c release was markedly potentiated by the inhibitors (Figure 2f). Similar increases were observed for caspase-3 activity in B-ALL cell lines co-treated with Dex and inhibitors compared to Dex alone (Figure 2g). Consistent with our previous findings [17], the potentiating effect of Dex-mediated mitochondrial dysfunction with PLC pathway inhibition may not depend on mitochondrial Ca^2+^ load. Indeed, as shown in Figure 3a, measurement of mitochondrial Ca^2+^ indicated that the inhibitors notably abolished Dex-mediated mitochondrial Ca^2+^ release. The enzymatic activity of PLC is known to hydrolyze membrane phosphatidylinositol 4,5-bisphosphate (PIP_2_) to inositol 1,4,5-triphosphate (IP_3_) [22]. Upon binding of IP_3_, the receptor channel IP_3_R opens to allow the release of Ca^2+^ from endoplasmic reticulum stores into the cytosol. We then used an inhibitor of IP_3_-induced Ca^2+^ release, 2-APB, and observed similar increases in Δψ_m_ dissipation (Figure 3b,c) and ROS production (Figure 3d) in B-ALL cell lines treated with 2-APB in combination with Dex. Taken together, these data suggest that inhibition of the PLC pathway improves Dex sensitivity in B-ALL cells by altering mitochondrial function.

### 2.3. CXCR4 Inhibition Enhances Dex-Induced Mitochondrial Pathway Alteration in B-ALL Cells

**Figure 4:**
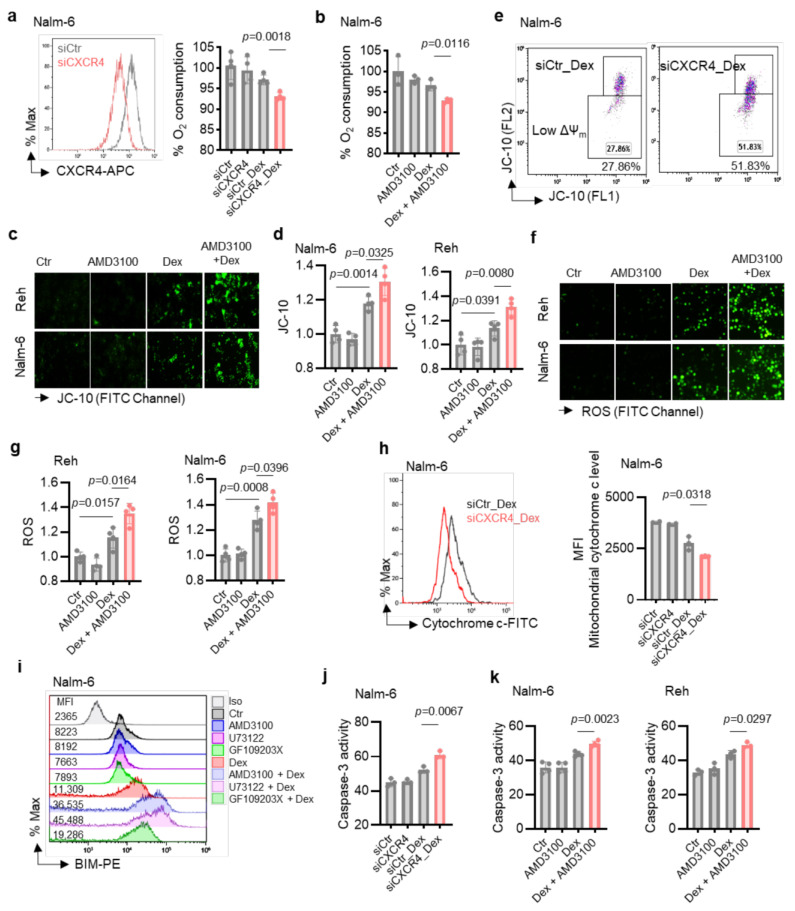
Co-treatment with Dex and CXCR4 siRNA or CXCR4 inhibitor AMD3100 markedly increases mitochondrial dysfunction in B-ALL cells. (**a**) Left: Representative FACS analysis of siRNA-transfected B-ALL cells. Right: Quantification of oxygen consumption rates of transfected cells and stimulated with 100 nM Dex for 24 h. (**b**) Oxygen consumption in cells stimulated with 100 nM Dex and/or 25 µM AMD3100 for 24 h. (**c**,**d**) Cells were exposed to 100 nM Dex in absence or presence of CXCR4 antagonist (AMD3100, 25 µM) for 24 h. (**c**) Images acquired with fluorescence microscope after JC-10 staining. Scale bar: 10 µm. (**d**) Fluorescence intensity for mitochondrial membrane potential changes was normalized to Ctr. (**e**) Representative flow cytometry plots of Nalm-6 cells transfected with either siCtr or siCXCR4 then stimulated with 125 nM Dex for 24 h, measuring mitochondria membrane potential change (ΔΨ_m_). (**f**,**g**) Cells were exposed for 24 h to 100 nM Dex in absence or presence of CXCR4 antagonist (AMD3100, 25  µM). (**f**) Images acquired with fluorescence microscope after DHR 123 staining. Scale bar: 10 µm. (**g**) Fluorescence intensity for ROS production was normalized to Ctr. (**h**) Representative flow cytometry plots and MFI of mitochondrial cytochrome c level of cells transfected with either siCtr or siCXCR4 then stimulated with 125 nM Dex for 24 h. (**i**) Intracellular BIM expression measured by flow cytometry. Cells were exposed to 100 nM Dex in absence or presence of 25 µM AMD3100, 1 μM U73122, or 1 μM GF109203X for 24 h. (**j**,**k**) Caspase-3 activity in siRNA-transfected cells after 24 h incubation with 100 nM Dex (**j**) or in cells exposed to 100 nM Dex in absence or presence of 25 µM AMD3100 (**k**). Unpaired two-tailed Student’s *t*-test, *n* = 3–4. Data are represented as mean ± SEM.

Since our in vitro results showed that the PLC pathway, activated by Dex via CXCR4 [19], strongly regulated mitochondrial function and Dex sensitivity, we next examined whether the effect of CXCR4 inhibition on Dex-induced B-ALL apoptosis [19] was mediated through the modulation of mitochondrial functions. To this end, we negatively regulated the expression of CXCR4 using small interfering RNA (siRNA) in Nalm-6 cells (Figure 4a, left) and evaluated mitochondrial respiration by measuring the OCR in siCXCR4 B-ALL cells. We found that Dex markedly decreased OCR in siCXCR4 B-ALL cells (Figure 4a, right). Consistent with these results, OCR induced by Dex were markedly reduced by the CXCR4 antagonist AMD3100 (Figure 4b). We next asked whether CXCR4 inhibition could affect Dex-mediated mitochondrial membrane potential (Δψ_m_) changes in B-ALL cells using JC-10 staining, a lipophilic cationic dye. We observed a significant increase in Δψ_m_ depolarization in CXCR4 antagonist (AMD3100)-treated B-ALL cells compared to Ctr cells in response to Dex stimulation (Figure 4c,d). The ability of CXCR4 inhibition to increase Δψm depolarization in the presence of Dex was further confirmed in CXCR4-silenced B-ALL cells (Figure 4e). Moreover, a significant increase in ROS production (Figure 4f,g) was observed after co-treatment of B-ALL cells with Dex and the CXCR4 antagonist. The production of ROS and the Δψ_m_ collapse led to the initiation of the mitochondria-mediated cell apoptosis cascade, involving cytochrome c release, Bcl-2 apoptotic protein activation, and caspase-3 activity. We then assessed whether the effect of CXCR4 pathway inhibition on Dex-induced apoptosis was associated with the mitochondrial release of cytochrome c, Bcl-2 apoptotic protein, and caspase-3 activity. As expected, CXCR4-silenced B-ALL cells showed greater mitochondrial cytochrome c release than control cells (siCtr) when treated with Dex (Figure 4h). Considering the apoptotic function of BIM and its importance in Dex-induced apoptosis in B-ALL cells [23], we subsequently analyzed BIM expression. Interestingly, we found that BIM was upregulated in response to Dex alone and co-treatment with PLC, PKC, and CXCR4 signaling inhibitors potentiated this effect (Figure 4i). This apoptotic pathway was further confirmed by a significant increase in caspase-3 activity in CXCR4-silenced B-ALL cells treated with Dex (Figure 4j), as well as in the presence of CXCR4 antagonist AMD3100 (Figure 4k). These data, along with the results obtained above, suggest that inhibition of the CXCR4/PLC axis contributes to the enhancement of the apoptotic effect of Dex through mitochondrial dysfunction.

## 3. Discussion

The present study expands our previous research regarding the CXCR4/PLC axis in B-ALL sensitivity to GCs [17,19], demonstrating its functional interaction with ROS homeostasis and mitochondrial cell death pathways. Treatment of B-ALL cells with inhibitors of this axis resulted in increased Dex sensitivity through ROS accumulation, decreased O_2_ consumption, Δψ_m_ collapse, cytochrome c release, caspase-3 activation, and BIM release.

The initial response to GC therapy in B-ALL is a key predictor of how patients will respond to chemotherapy and their long-term outcomes. While much research has focused on the mechanisms of acquired drug resistance during B-ALL treatment, our recent study highlights the importance of non-genomic GC responses in B-ALL, where GCs paradoxically induce their own resistance by activating CXCR4 signaling, which in turn activates the PLC pathway [19]. This process increases cytosolic Ca^2+^ levels and triggers protein kinase C (PKC) signaling, promoting the survival of B-ALL cells. Inhibition of the CXCR4/PLC axis significantly overcame Dex resistance in B-ALL cell lines (both in vitro and in vivo), as well as in cells from Dex-resistant ALL patients [19]. Drug resistance is linked to various factors, with mitochondrial dysfunction being one of them [24,25,26]. Targeting mitochondria and ROS has emerged as a strategy in cancer therapy, and several studies have confirmed their potential as a treatment for ALL patients [27,28,29,30,31,32]. Therefore, targeting CXCR4/PLC could influence mitochondrial function in B-ALL, potentially preventing drug resistance in B-ALL cells.

Our study demonstrated that the PLC pathway plays a crucial role in the metabolic reprogramming of B-ALL cells by regulating mitochondrial function and cell death in response to oxidative stress. These mitochondrial processes in response to Dex in B-ALL cells could be enhanced by the inhibition of the CXCR4/PLC axis, suggesting that increased Dex sensitivity is linked to mitochondria-mediated pro-apoptotic factors. Supporting this model, our previous results showed that co-treatment with Dex and a TRPC3 inhibitor or cytosolic Ca^2+^ chelator significantly increased Δψ_m_ collapse, ROS production, cytochrome c release, and caspase-3 activity in B-ALL cells [17,18,33]. Consistently, a recent study demonstrated that Dex releases BIM protein, which is associated with the permeabilization of the mitochondrial outer membrane, a point of no return for mitochondrial apoptosis [34]. Our data therefore suggest that CXCR4/PLC-induced Dex resistance may protect B-ALL cells from the mitochondrial apoptosis pathway. This supports the potential for further evaluation of CXCR4/PLC axis inhibitors for clinical use in B-ALL.

## 4. Materials and Methods

### 4.1. Cell Lines and Reagents

The B-ALL cell lines Nalm-6 and Reh (Deutsche Sammlung von Mikroorganismen und Zellkulturen, DSMZ^®^, Braunschweig, Deutschland) were cultured in RPMI 1640 medium (Eurobio^®^, Courtaboeuf, France) containing 10% fetal bovine serum (FBS, Eurobio^®^), 2 mM of L-glutamine (Eurobio^®^) with 5000 UI/L penicillin and 50 mg/L streptomycin (Eurobio^®^). B-ALL cell lines were maintained at 37 °C in a 5% CO_2_ humidified atmosphere. All cell lines were authenticated and routinely tested for mycoplasma contamination using the MycoAlert PLUS detection kit (Lonza, LT07-705, Saint-Cyr-l’École, France). Rhod-2/AM, U73122, AMD3100, and GF109203X were purchased from Abcam Biochemicals, Cambridge, UK. Dimethylsulfoxide (DMSO), 2-APB (2-aminoethoxydiphenyl borate), and dexamethasone were purchased from Sigma Aldrich, Saint-Quentin-Fallavier, France. In this study, we used two concentrations of Dex (100 nM and 125 nM) depending on the experimental conditions, in order to validate the robustness of the response

### 4.2. LIVE/DEAD^®^ Viability/Cytotoxicity Kit Assay

The viability of treated B-ALL cells was assessed by using the LIVE/DEAD^®^ Cell Vitality Assay (Abcam, Cambridge, UK) as previously described [19]. Analysis was performed under LSR-Fortessa (BD Biosciences, Le Pont de Claix, France) flow cytometry using Kaluza software (A85810 AB).

### 4.3. Caspase-3 Activity

Ac-DEVD-AFC substrate (Enzo Life Sciences (ELS) AG, Villeurbanne, France) was used to measure caspase-3 activity, according to the manufacturer’s instruction. Briefly, cells were treated with or without test compounds for 24 h, and then collected and lysed in cell lysis buffer (HEPES 50 mM, NaCl 100 mM, DTT 10 mM, CHAPS 0.1%, EDTA 1 mM, pH 7.4). Cell lysate (20 µg) was added to 100 μL of caspase-3 buffer containing 40 μM of the caspase-3 substrate Ac-DEVD-AFC (fluorogenic) as a final concentration, and incubated at 37 °C for 1 h in the dark. Caspase-3 activity was assessed by measuring fluorescence (Ex 400 nm/Em 505 nm) using an SAFAS Xenius XC Spectrofluorometer (MC 98000, Monaco) or Flexstation-3 Molecular Devices (Winnersh, UK).

### 4.4. Flow Cytometry Measurement of Cell-Surface CXCR4

Antibodies against human (Biolegend, 306510, Paris, France) APC-conjugated anti-CXCR4 or isotype control APC-Mousse IgG 1k (BD Biosciences) were used. Cell-surface CXCR4 expression was measured on flow cytometer and analyzed using Kaluza software.

### 4.5. Short Interfering RNA Knockdown of CXCR4

Silencing experiments were performed with ON-TARGETplus human siRNA (25 nM) targeting CXCR4 (Horizon-Dharmacon, J-005139-08, Cambridge, UK) or non-targeting siRNA as a control (Horizon-Dharmacon, D-001810-10). siRNAs were introduced into ALL cells using DharmaFECT Transfection Reagent (Dharmacon, T-2001, Cambridge, UK) according to the manufacturer’s instructions. After a 24 h incubation, the medium was aspirated and replaced with fresh medium without siRNA for 48 h. The transfected ALL cells were then used for various experiments and analyses.

### 4.6. Measurement of Pro-Apoptotic BIM Level by Flow Cytometry

ALL cells were stimulated with or without test compounds, and levels of the pro-apoptotic molecule BIM was assessed by intracellular staining flow cytometry [35]. After fixation with buffer (Biolegend, cat. no. 420801) and permeabilized with True-Phos Perm Buffer (Biolegend, cat. no. 425401), cells were stained PE anti-BIM (Santa Cruz Biotechnology, sc-374358). Flow cytometry data were collected on a BD LSRFortessa, and analyzed with Kaluza software.

### 4.7. Mitochondrial Membrane Potential (ΔΨm)

ALL cell lines were incubated in cultured medium with or without test compounds for 24 h before loading with 20 µM of JC-10 (a fluorescent ΔΨm dye, Santa Cruz Inc., Heidelberg, Germany) for 30 min at 37 °C. After washing, images were acquired with a Zeiss Axiovert 200M fluorescence microscope using the FITC channel and the fluorescence intensity for both J-aggregates and monomeric forms of JC-10 was measured using an SAFAS Xenius XC Spectrofluorometer (MC 98000, Monaco) at Ex 485 nm/Em520. Alternatively, ΔΨm was assessed by flow cytometry and analyzed using Kaluza software.

### 4.8. Intracellular Reactive Oxygen Species (ROS)

ROS production was determined using a cell-permeable fluorogenic probe, dihydrorhodamine 123 (DHR 123) (Santa Cruz Inc., Heidelberg, Germany). DHR 123 is oxidized by cellular peroxides to highly fluorescent rhodamine 123. ALL cell lines were treated with or without test compounds for 24 h and then loaded with 20 µM of DHR 123 for 30 min at 37 °C. After washing, images were acquired with a Zeiss Axiovert 200M fluorescence microscope (Oberkochen, Germany) using FITC channel and the fluorescence intensity was monitored with an SAFAS Xenius XC Spectrofluorometer (MC 98000, Monaco) using Ex 500 and Em 536 nm wavelengths.

### 4.9. Cytochrome c Release

B-ALL cell lines were treated with or without test compounds for 24 h. Cytochrome c was detected by a FlowCellectTM Cytochrome c kit (Millipore, Guyancourt, France) according to the manufacturer’s protocol. Briefly, cell mitochondria were permeabilized and fixed according to the protocol soon after harvesting and then incubated for 30 min with 10 µL of anti-Cytochrome c-FITC antibody in the dark at RT. After completion of the staining process, blocking buffer was used to wash the cells, which were then centrifuged for 5 min at 400× *g*. Then, 200 µL of blocking buffer was used to resuspend the cell pellet. The fluorescence expression of the samples was then analyzed by flow cytometer LSRFortessa cell analyzer (BD Biosciences) and analyzed using Kaluza software. Viable cells demonstrate higher levels of cytochrome c staining, whereas apoptotic cells which have released their cytochrome c from the mitochondria to the cytoplasm demonstrate reduced staining intensity when probed with an anti-cytochrome c FITC antibody. Alternatively, cytosolic cytochrome c was assessed using the ELISA kit (no: ab110172, Abcam Biochemicals, Paris, France) according to the manufacturer’s instructions.

### 4.10. Oxygen Consumption Assays

ALL cells were treated with or without test compounds alone or in combination for 24 h. Extracellular oxygen consumption (OCR) was measured by an MITO-ID^®^ Extracellular O2 Sensor Kit, High Sensitivity (Enzo Life Sciences, cat. no. ENZ-51045-K100) according to the manufacturer’s protocol.

### 4.11. Gene Set Enrichment Analysis (GSEA) of RNA-Sequencing Data

Analysis was performed as previously described [19] using a GSEA analysis tool http://www.broadinstitute.org/gsea/index.jsp (accessed on 11 October 2023). RNA-Seq data were extracted from our previous data set [19] available in the Gene Expression Omnibus (GEO) database under access number GSE214990.

### 4.12. Measurement of Mitochondrial Ca^2+^ Response, [Ca^2+^]m

To assess mitochondrial calcium levels, acute lymphoblastic leukemia (ALL) cells were incubated with the cell-permeable calcium indicator Rhod-2/AM (2 µM, dissolved in DMSO) in RPMI medium for 1 h at 37 °C. Following incubation, cells were washed three times with Rhod-2-free medium to remove excess dye. Mitochondrial calcium fluorescence was then measured by exciting Rhod-2 at 552 nm and recording the emitted fluorescence at 581 nm. 

### 4.13. Data and Statistical Analysis

Statistical analyses were conducted using GraphPad PRISM Software (v 8.0.2) using two-tailed unpaired Student’s *t*-test. Differences were considered statistically significant at *p* < 0.05. Data are expressed as mean ± S.E.M.

## Data Availability

The analyzed data and original contributions in this study are included in the article. Further inquiries can be directed to the corresponding author.
